# Electrochemistry of Organic and Organometallic Compounds

**DOI:** 10.3390/molecules31030535

**Published:** 2026-02-03

**Authors:** Angel A. J. Torriero

**Affiliations:** School of Life and Environmental Sciences, Faculty of Science, Engineering & Built Environment, Deakin University, Burwood, VIC 3125, Australia; angel.torriero@deakin.edu.au; Tel.: +61-3-9244-6897

## 1. Introduction

Electrochemistry occupies a distinctive position within modern chemistry by providing a direct and controllable link between molecular structure, redox behaviour, and functional performance. In organic and organometallic chemistry, electrochemical methods now extend well beyond their traditional analytical role, underpinning mechanistic elucidation, materials development, catalysis, sensing, and emerging sustainable synthetic strategies [1,2,3,4]. Through precise control of electron transfer and access to transient redox states, electrochemistry has become an enabling framework for integrating molecular design with functional response across diverse chemical systems [2,3,4,5].

The Special Issue “Electrochemistry of Organic and Organometallic Compounds” was conceived to capture this evolution by showcasing research that integrates electrochemical methodologies with molecular design, spectroscopy, theory, and application-driven objectives. The six contributions collected in this issue reflect the breadth and maturity of the field, spanning spectroelectrochemical insights into organometallic catalytic systems, functional electroactive films and emitters, foundational review-level consolidation in organic redox chemistry, and a clinically relevant point-of-care biosensing platform [6,7,8,9,10,11]. Collectively, these works illustrate how electrochemistry continues to expand both its conceptual reach and its practical relevance.

## 2. Themes and Contributions Across the Special Issue

A prominent theme emerging from this Special Issue is the central role of structure–property relationships in governing electrochemical behaviour. Studies centred on coordination complexes show how ligand architecture, metal centre selection, and electronic coupling determine redox accessibility and functional response. Zowiślok and coworkers present iridium(III) complexes incorporating functionalised terpyridines and bis(thiazolyl)pyridines, linking structure with photophysical behaviour supported by rigorous characterisation [6]. In a complementary direction, Barbero and coworkers provide spectroelectrochemical insights into manganese and rhenium bipyridine complexes relevant to carbon dioxide electroreduction, highlighting how combined electrochemical and spectroscopic approaches clarify redox processes and the formation of intermediates in catalytically relevant systems [7]. Together, these studies reinforce a broader trend in organometallic electrochemistry: a shift away from reporting redox potentials in isolation toward constructing integrated mechanistic narratives. These narratives are increasingly supported by complementary experimental techniques [4,5,12].

Spectroelectrochemistry emerges in this issue not as an optional add-on, but as a methodological bridge between electrochemical observables and functional chemical states. For molecular catalysts and photoactive complexes, redox events often involve overlapping electronic transitions and short-lived intermediates, making combined electrochemical and spectroscopic interrogation essential [4,5,12]. The contributions presented here demonstrate how mechanistic clarity is enhanced when electrochemical control is paired with real-time optical readouts [7,12].

A second major pillar of the Special Issue is the development of organic and organometallic systems designed for electroactive optical functionality. Zhou and co-workers report a diboron thermally activated delayed fluorescence emitter and examine its electrochemiluminescence behaviour, demonstrating how molecular design choices translate into electrochemically driven emission outcomes [8]. Gu and co-workers develop a near-infrared electrochromic film by oxidative electropolymerisation of a triphenylamine modified terpyridine platinum(II) chloride, achieving high optical contrast and stability and underscoring how polymer growth conditions and redox stability influence device-relevant performance [9]. These papers illustrate a practical point: for electroactive optical materials, the useful question is often not whether a redox process occurs, but whether it is reversible, stable, and optically efficient enough to support a real operational cycle [3,10,13].

The Special Issue also includes a review that consolidates and clarifies the electrochemistry of a widely studied class of bioactive organics. Naróg and Sobkowiak review the electrochemistry of flavonoids, summarising oxidation behaviour and structure–activity relationships, and highlighting persistent challenges in comparability across electrodes, media, and analytical protocols [10]. This contribution is valuable because it foregrounds a problem that extends far beyond flavonoids: electrochemical interpretation is often highly sensitive to experimental context, yet reporting conventions and data normalisation remain inconsistent across subfields [2,3,10]. Addressing this gap is important not only for fundamental understanding but also for translation into analytical and diagnostic settings.

Beyond materials and mechanistic electrochemistry, the Special Issue demonstrates direct clinical relevance through the application of electroanalysis. Mruthunjaya and coworkers report an electrochemical disposable biosensor for monitoring dabigatran for point-of-care anticoagulation therapy [11]. This work exemplifies how electrochemical principles can be translated into practical formats that address constraints such as speed, simplicity, and deployment in settings outside specialist laboratory infrastructure. It also highlights a broader direction for the field: as electrochemical sensing platforms mature, performance metrics increasingly need to include usability, robustness, and context-specific reliability, rather than solely analytical sensitivity [2,11,14].

## 3. Reflections on the Evolving Role of Electrochemistry in Molecular Design

Taken together, the contributions in this Special Issue illustrate a clear shift in how electrochemistry is positioned within contemporary chemical research. Electrochemical methods are increasingly integrated into the early stages of molecular design and functional optimisation, rather than being confined to post hoc characterisation or mechanistic confirmation. This shift is evident across both organic and organometallic systems, where redox behaviour, stability, and electrochemical addressability are treated as design parameters rather than secondary properties [3,4,8,9].

At the same time, this evolution introduces new conceptual challenges. As electrochemistry becomes embedded across materials science, catalysis, and bioanalysis, the expansion of experimental platforms and performance metrics reflects both the maturity and breadth of the field. This diversity enables innovation, but it also demands greater care in contextualising and interpreting electrochemical results across subdisciplines. The works presented in this Special Issue highlight the value of complementary approaches, particularly the integration of electrochemical, spectroscopic, and theoretical methods, in addressing these challenges [5,7,12]. Moving forward, a stronger emphasis on methodological transparency, reporting completeness, and the contextualisation of electrochemical data will be essential if molecular-level insights are to translate reliably into functional performance [2,3,4,10,15].

## 4. Sustainability and Interdisciplinarity as Emerging Drivers

A further theme emerging implicitly across this Special Issue is the increasing influence of sustainability and interdisciplinarity in shaping electrochemical research. Electrochemical methodologies offer intrinsic advantages aligned with sustainable chemistry, including precise control over reaction pathways, reduced reliance on stoichiometric reagents, and direct compatibility with renewable electricity inputs [1,2,3,15]. In molecular and materials electrochemistry, these attributes enable reaction and device optimisation to be guided by measurable redox efficiency, reversibility, and stability, rather than by yield alone. As electrification becomes a practical lever for decarbonising chemical innovation, such control positions electrochemistry not merely as a greener alternative, but as a framework for sustainability-driven design [3,15].

In parallel, many of the challenges tackled by contemporary electrochemistry now sit at disciplinary boundaries. Progress in electrochromic films and electrochemiluminescent emitters requires close interaction between synthetic chemistry, electroanalysis, photophysics, and device engineering [8,9,13]. Translation of electrochemical sensors into biomedical and point-of-care settings requires engagement with clinicians, regulatory considerations, and user-centred design constraints [11,14]. The diversity of contributions in this Special Issue reflects this interdisciplinary reality and underscores that future advances will be accelerated when chemical insight is paired with engineering rigour and application context [3,11,14].

## 5. Gaps, Challenges, and Future Directions

Despite the progress reflected in these contributions, several persistent challenges remain evident. Establishing robust correlations between electrochemical observables and functional performance remains a central challenge, particularly for complex organometallic and conjugated organic systems, where stability, mass transport, electrode interactions, and coupled chemical reactions complicate interpretation [2,3,4,5,12]. These limitations are compounded by persistent issues of long-term redox stability and operational durability in device-facing materials, where performance is often governed by cycling behaviour rather than single-scan responses [8,9,13]. In analytical electrochemistry, data comparability and mechanistic assignment also depend on the electrode material, surface history, medium composition, and scan protocol, underscoring the need for stronger reporting standards and more consistent benchmarking [2,10].

Looking forward, future research is likely to be shaped by converging methodological and translational trends. Increased emphasis on operando and in situ approaches will enable more realistic assessment of electrochemical systems under working conditions and accelerate mechanistic refinement [4,5,12]. Advances in modelling and data interpretation frameworks will help bridge molecular-level insight and macroscopic behaviour, improving predictive capability and design efficiency [4,5,12]. At the same time, the integration of electrochemical systems into functional devices, sensors, and energy-related technologies will demand closer collaboration among chemists, materials scientists, and engineers, alongside an increased focus on standardised performance metrics that match end-use conditions [3,11,13,14].

## 6. Conclusions

In closing, this Special Issue captures the diversity, sophistication, and ongoing evolution of electrochemistry within organic and organometallic chemistry. The contributions presented here advance mechanistic understanding, demonstrate the rational design of electroactive functional materials, consolidate key knowledge in organic redox chemistry, and illustrate their translation into clinically relevant point-of-care electroanalysis [6,7,8,9,10,11]. It is hoped that this Special Issue will not only stimulate further discussion and foster new collaborations but also serve as a snapshot of current advances while encouraging continued innovation at the interface of molecular design, electrochemical insight, and functional application.
